# Spontaneous Migration of a Ventriculoperitoneal Shunt into the Venous System: A Multidisciplinary Approach

**DOI:** 10.7759/cureus.7779

**Published:** 2020-04-22

**Authors:** Megan M Finneran, Emilio Nardone, Dario A Marotta, Glen B Smith, Ajeet Gordhan

**Affiliations:** 1 Neurosurgery, Advocate BroMenn Medical Center, Normal, USA; 2 Neurosurgery, Central Illinois Neuroscience Foundation, Bloomington, USA; 3 Department of Research, Alabama College of Osteopathic Medicine, Dothan, USA; 4 Department of Neurology, Division of Neuropsychology, University of Alabama, Birmingham, USA; 5 Cardiothoracic Surgery, OSF St. Joseph Medical Center - OSF Healthcare, Bloomington, USA; 6 Neurointerventional Radiology and Surgery, OSF St. Joseph Medical Center - OSF Healthcare, Bloomington, USA

**Keywords:** ventriculoperitoneal shunt, catheter migration, venous system, cardiopulmonary vasculature

## Abstract

Ventriculoperitoneal shunt catheter migration is a rare but documented complication. The exact mechanism of this occurrence is not well understood. We report the case of an 81-year-old male who initially presented with symptoms consistent with normal pressure hydrocephalus. A ventriculoperitoneal shunt was placed uneventfully. Four months later, the patient presented complaining of a persistent headache despite multiple adjustments in the shunt setting. Shunt series radiographs demonstrated the distal catheter passing through the superior vena cava and looping into the right cardiac atrium and ventricle. Catheter retrieval was attempted from a proximal retroauricular incision but required a combination of snare technique by interventional radiology and, ultimately, surgical venotomy by a cardiothoracic surgeon. The distal catheter was replaced in the abdomen, and the patient had no further complications. This case is the first of its kind reported in the literature that includes a treatment team comprising neurosurgery, interventional radiology, and cardiothoracic surgery. We highlight the importance of a multidisciplinary approach to best address the migrated catheter.

## Introduction

Ventriculoperitoneal (VP) shunt placement is a common neurosurgical procedure that occurs around 30,000 times per year in the United States [1]. VP shunting is most frequently used to treat hydrocephalus. Persons treated for normal pressure hydrocephalus have better functional outcomes and lower complication rates than those treated for other indications [2]. Nevertheless, VP shunt complication rates remain relatively high. The incidence of VP shunt malfunctions requiring surgical revision occurs in approximately 15% of adult patients with VP shunts and is associated with increased age, prolonged hospital stays, and lower Glasgow coma scales [3]. The majority of complications result from overdrainage of cerebrospinal fluid (CSF), which can usually be managed with nonsurgical valve adjustments [4]. Infections, bleeding, CSF leak, and shunt migration are also known VP shunt complications. In this case, we report the intracardiopulmonary migration of a distal VP shunt catheter in an 81-year-old man. We used a multidisciplinary approach involving interventional radiology, cardiothoracic surgery, and neurological surgery to strategically revise the placement of the migrated shunt.

## Case presentation

History and examination

An 81-year-old male with an extensive medical history, including lung cancer status post-lobectomy, restless leg syndrome, depression, and peripheral artery disease, presented to the neurosurgical clinic for evaluation of frequent falls. The patient reported backward falls with associated dizziness, confusion, and memory issues. He denied problems with urination. Magnetic resonance imaging (MRI) and computed tomography (CT) of the head showed frontotemporal atrophy and ventriculomegaly that was out of proportion for the atrophy. A neurologist had diagnosed the patient with normal pressure hydrocephalus. Gait was unsteady, but otherwise he had no neurological deficits on examination.

A right-sided VP shunt with a Codman Hakim programmable valve (Integra LifeSciences Corporation, Princeton, NJ, USA) was placed without complication. The shunt pressure was set to 12 cm H_^2^_O. The patient was discharged home on post-operative day 1 with no neurological deficits.

**Figure 1:**
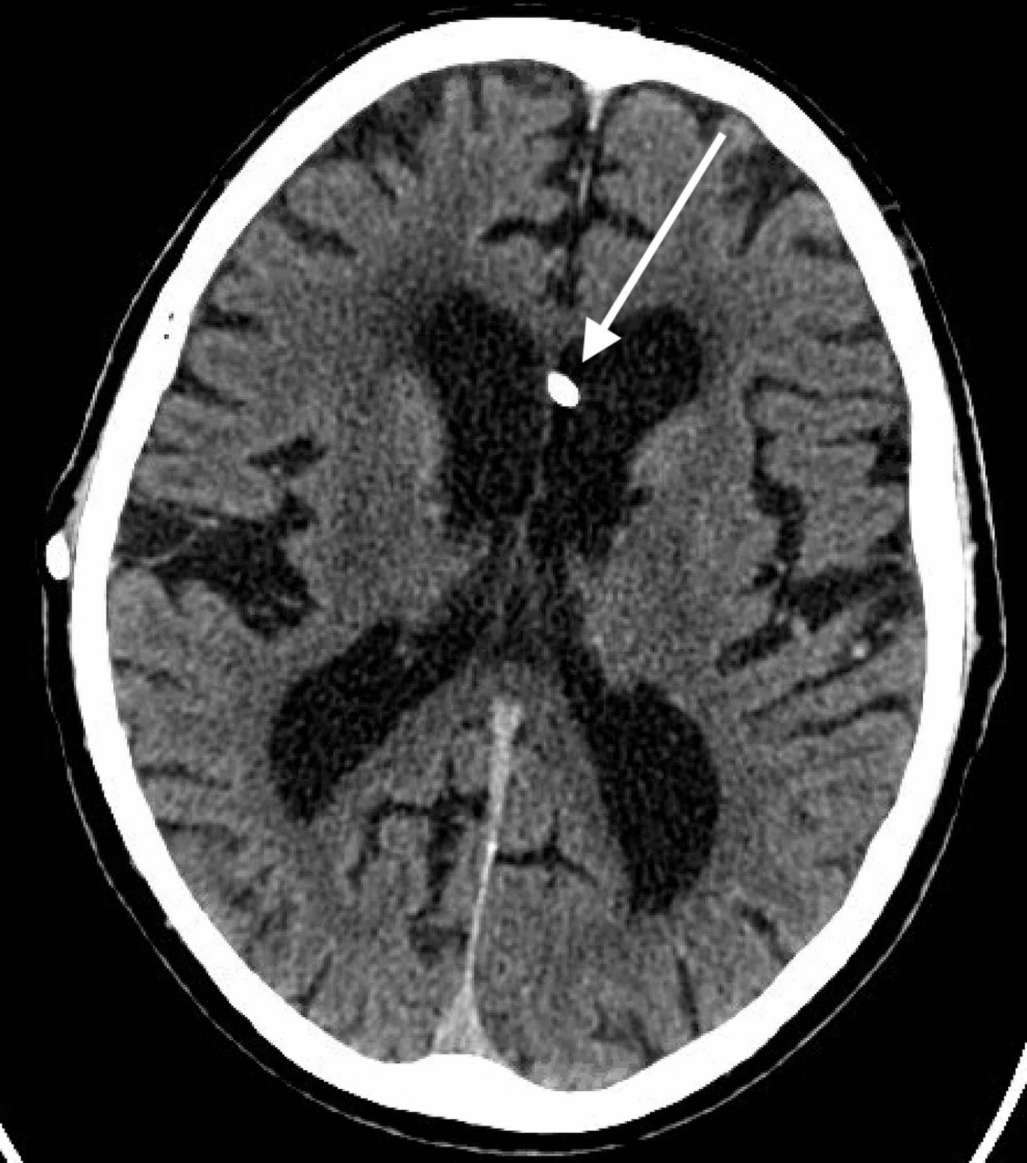
Computed tomography (CT) of the head without contrast performed during workup for the patient’s headaches shows tip of the proximal catheter within the left lateral ventricle.

He presented to the office two weeks later with mild-to-moderate positional headache. CT of the head showed the catheter within the ventricular system (Figure 1).

The shunt setting was adjusted from 12 cm H_2_O to 14 cm H_2_O, which was confirmed on skull X-ray. His symptoms initially resolved, but the patient presented to the office one month later with bifrontal headache, nasal drainage, and congestion; he was undergoing a workup for sinusitis by his primary care provider. On physical examination, gait was steady and he had no neurological deficits. Three months post-operatively, the patient reported persistent headache.

**Figure 2:**
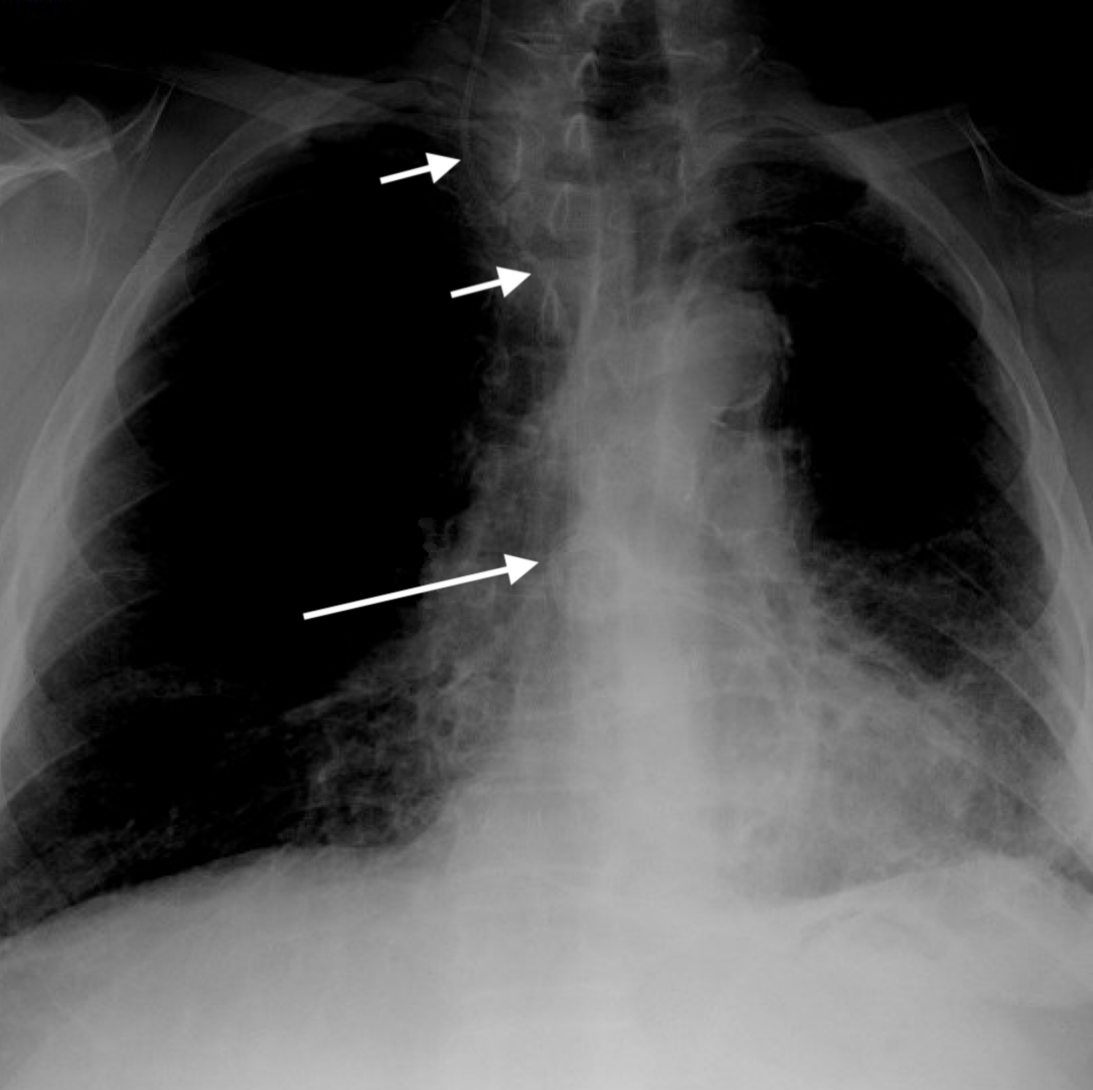
Anteroposterior chest X-ray performed as part of a shunt series demonstrates migration of the distal catheter of the ventriculoperitoneal shunt through the superior vena cava (short arrows) and coiling into the right heart (long arrow).

At that time, shunt series radiographs were performed and demonstrated migration of the distal aspect of the shunt catheter through the superior vena cava (SVC) into the right side of the heart (Figure 2).

Procedure

**Figure 3:**
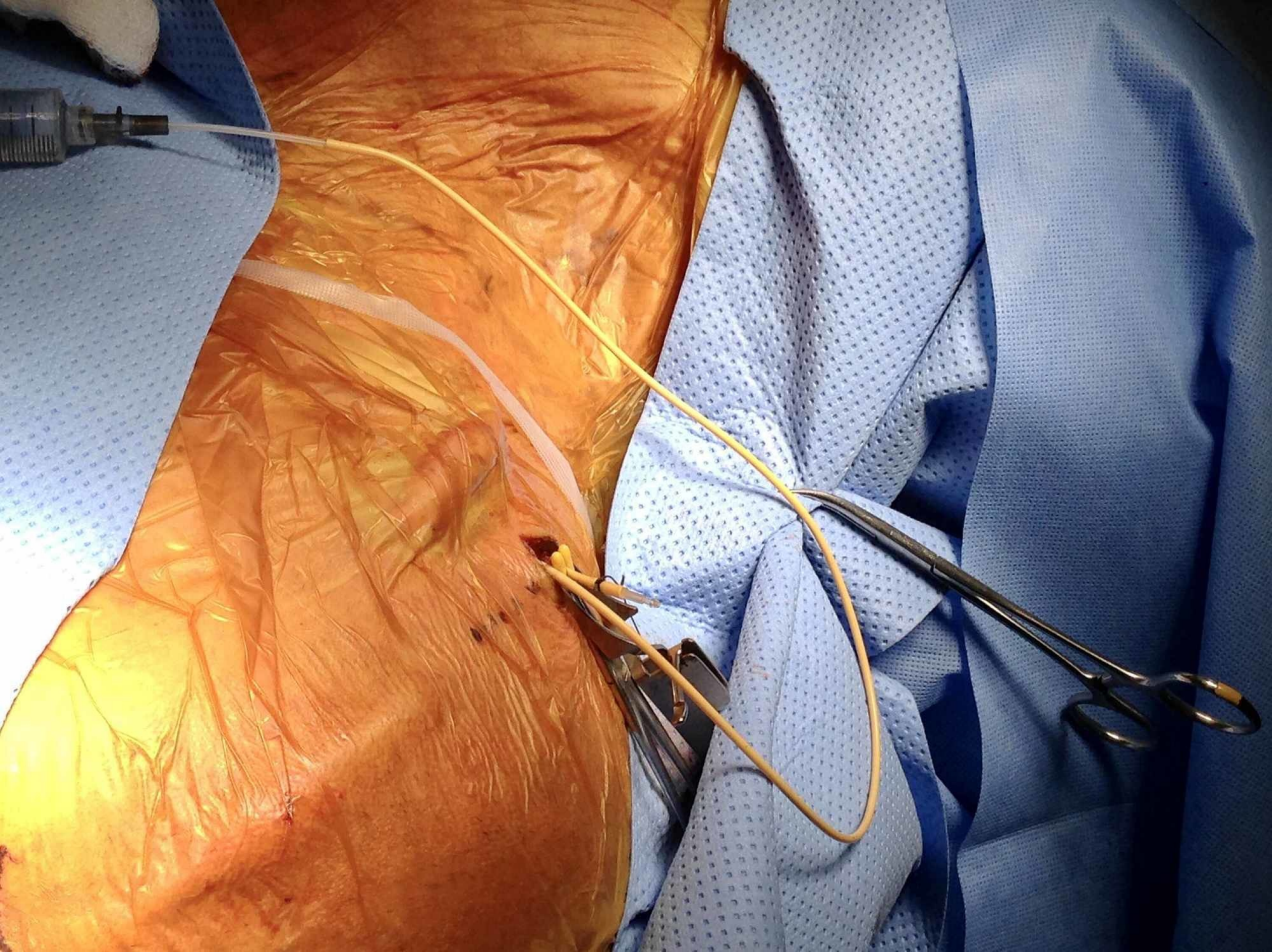
Intraoperative photo demonstrates right retroauricular incision with a portion of the distal shunt catheter removed.

Interventional radiology and cardiothoracic surgery services were consulted. An initial attempt was made to retrieve the migrated catheter proximally by the neurosurgical service. A small retroauricular incision was made, and Isovue-300® contrast (Bracco Diagnostics, Princeton, NJ, USA) was injected into the shunt catheter at the level of the incision using an angiocatheter. Opacification of the looped catheter was noted within the right atrium, right ventricle, and minimally within the right pulmonary artery segment. Proximal removal of the shunt catheter was attempted under fluoroscopic observation, and approximately 40 cm of tubing were extracted (Figure 3).

**Figure 4:**
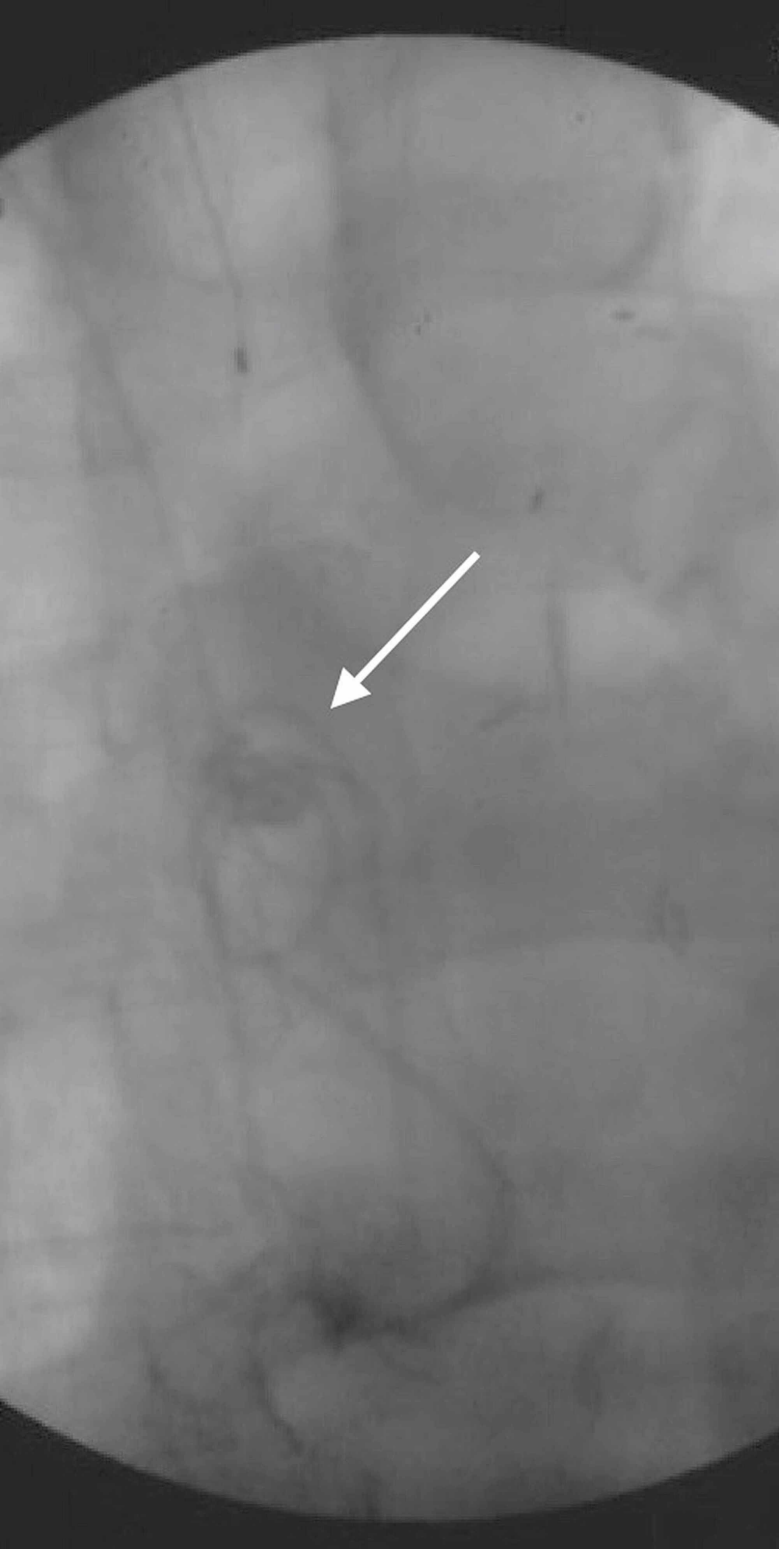
Fluoroscopy allows visualization of the catheter coiling in the superior vena cava (arrow) after a portion of the distal catheter had been removed. At this point, resistance was encountered and the attempt at proximal removal was aborted.

At that point, further removal was met with marked resistance, and additional coiling of loops could be seen within the SVC (Figure 4).

**Figure 5:**
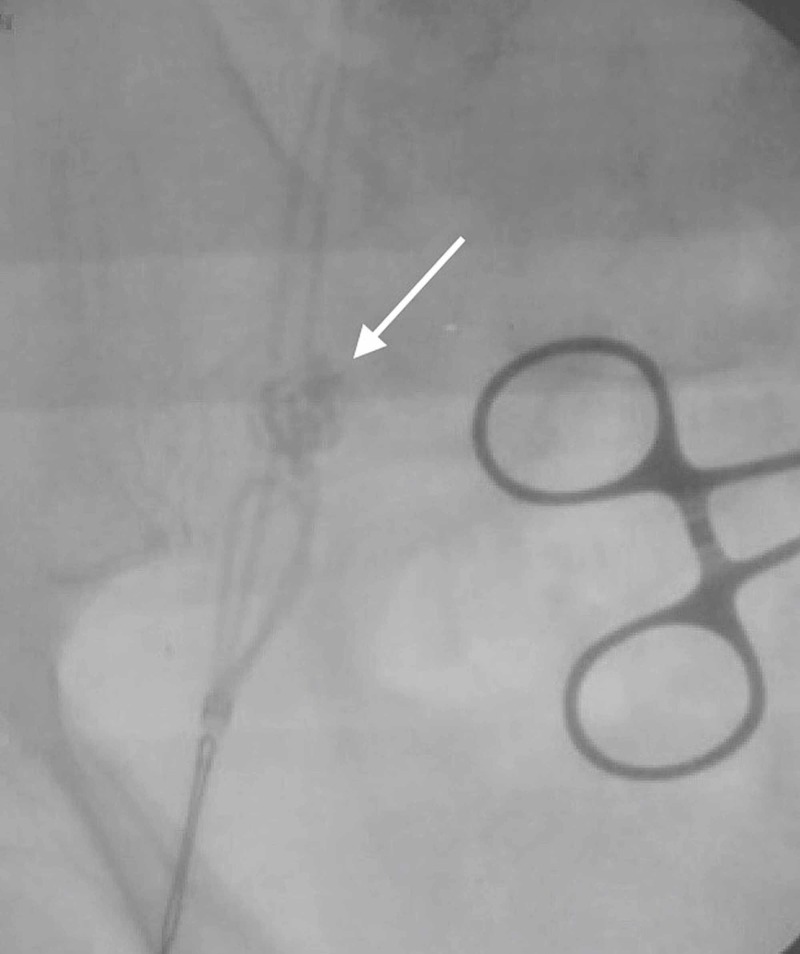
The right common femoral vein was accessed and a 2-mm Snare® delivery catheter was advanced over a Stork wire. Under fluoroscopic observation, the looped shunt catheter was snared and withdrawn to the right common femoral vein (arrow), where it was ultimately removed surgically through venotomy.

The right common femoral vein was then accessed by the interventional radiologist using a 19-gauge single-wall needle. A 7-French sheath was inserted over a J-tipped guidewire. Under fluoroscopic observation, a 5-French angled tapered glide catheter was inserted to the SVC through the right atrium. A 2-mm Snare® delivery catheter (Merit Medical, Jordan, UT, USA) was advanced through the right internal jugular vein over a Stork wire. Under fluoroscopic observation, the looped shunt catheter was snared and proximally withdrawn to the right common femoral vein (Figure 5).

**Figure 6:**
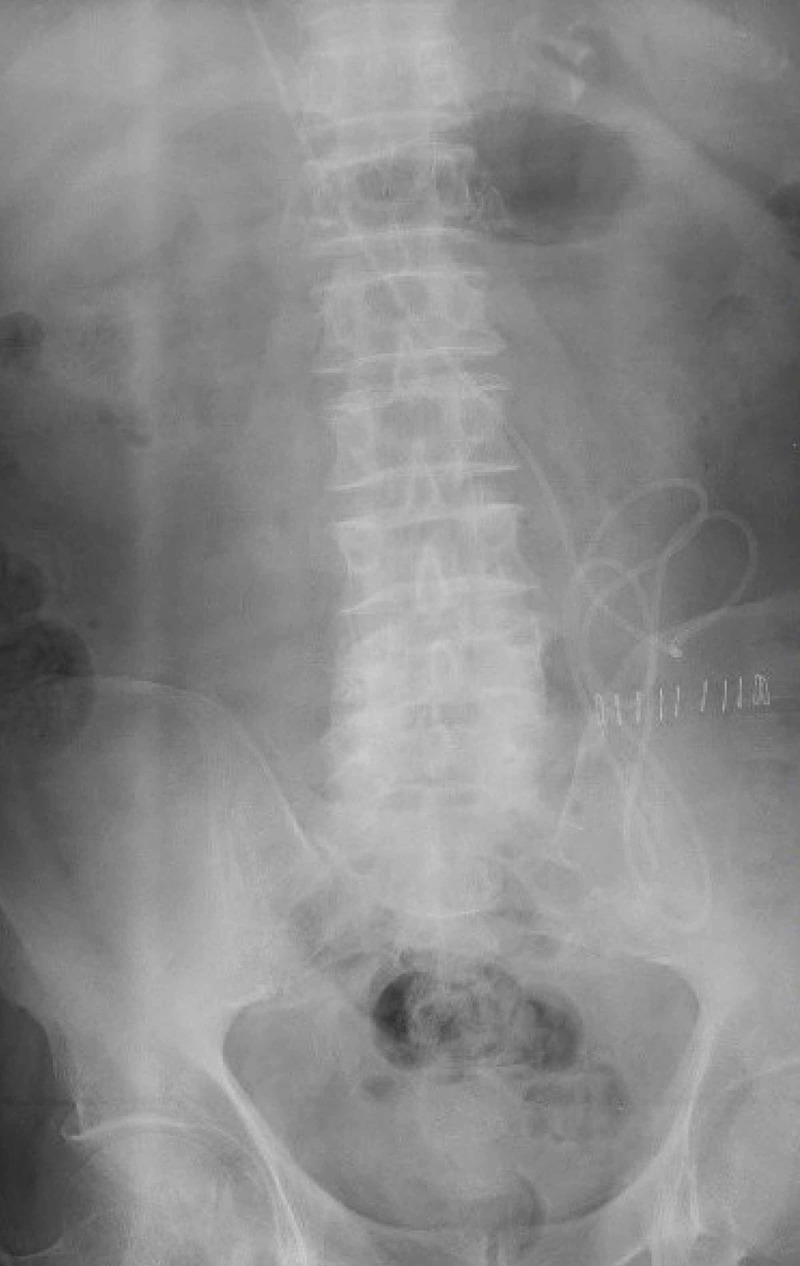
Post-operative abdominal X-ray confirms the location of distal catheter in the peritoneum.

Prior to withdrawal, a suture was attached to the proximal aspect of the shunt to retain proximal control. The shunt catheter was wedged within the proximal aspect of the right common femoral vein sheath. It was then surgically extracted through common femoral vein transverse venotomy by the cardiothoracic surgeon. The distal catheter was replaced, and placement was confirmed with an abdominal X-ray (Figure 6).

Post-operative course

The patient tolerated the procedure well and was extubated in the operating room. He was discharged home on post-operative day 1 with the shunt valve set to 11 cm H_2_O. He was seen in the office two months after surgery complaining of positional headaches, and the shunt was reset to 12 cm H_2_O. Thereafter, he had no further alterations to the valve setting or shunt catheter.

## Discussion

Roughly 55% of shunt revisions are unavoidable and typically occur after three months of implantation [5]. Preoperative symptoms such as headache, gait instability, cognitive decline, and urinary incontinence have been described in the literature as predictors of shunt malfunction [6]. Obesity and previous shunt surgery are more specific independent risk factors for catheter migration [7]. Distal catheter migration of VP shunts most commonly arises from peritoneal perforation and has been observed in a variety of organ systems such as lumbar soft tissue penetration following discectomy, bladder perforation and extrusion through the urethral orifice, ascension into the oropharyngeal cavity through the gastrointestinal tract, and descension into the scrotum through a congenital remnant [8-11]. Spontaneous intracardiac migration, such as the basis of this case report, has been documented previously in the literature, albeit rarely [12-17].

The exact mechanism surrounding distal catheter migration of this manner is unclear, but several hypotheses have been proposed. Two main theories have dominated the literature. One is that cannulation of the venous system occurs during distal tunneling of the shunt catheter that goes unnoticed intraoperatively [12,13]. The second is that the catheter, over time with repeated friction contact, erodes into an adjacent vein. This mechanism has historically been attributed to stiffness of the catheter [12]. The association between reduced occurrence of migration and softer, more flexible catheters suggests that a mechanical component exists in shunt migration [14]. In either case, the migration to the cardiopulmonary system is attributed to negative inspiratory pressure in the thoracic cavity that slowly draws the catheter into the vena cava and subsequently into the right atrium, right ventricle, and pulmonary arteries [15]. Apart from these, age has been shown to correlate with shunt migration, with a bimodal predilection in the early childhood and elderly populations [16]. Weak constitutions, specifically muscle and vasculature, diminish at each end of the aging spectrum and may serve as a predisposing factor.

Revision of VP shunts that spontaneously migrate into intracardiopulmonary system is often necessary to prevent serious complications. Intracardiac migration is conceptually similar to an unsecured ventriculoatrial (VA) shunt. Catheters that migrate into the cardiopulmonary system can become significantly scarred or tethered to cardiac tissue and may require an open heart surgery for extraction [17]. Further migration of the distal catheter past the atrium and into the pulmonary vasculature is problematic as it poses a risk of rapid cardiopulmonary destabilization [18].

Intraoperative fluoroscopy is a reliable method to assess and correct migrating VP shunt catheters and decreases early surgical revisions compared with controls without intraoperative radiography [19]. Interventional radiologists are particularly qualified to assist with endovascular retrieval of foreign bodies such as migrating catheters. For instance, utilization of transfemoral endovascular snaring, as in our case, has been successfully demonstrated in the past with a fragmented VA shunt [20]. However, due to the extensive coiling of catheter that occurred in our case, endovascular snaring was not sufficient to retrieve the catheter outright. After localization with the snare, a venotomy was required to safely withdraw the catheter. We suggest employing a multidisciplinary care team comprising a neurosurgeon, a cardiothoracic surgeon, and an interventional radiologist. This approach allows for flexibility in response to the uncertainty associated with the procedure.

## Conclusions

VP shunt migration into the cardiovascular structures presents unique challenges with surgical revision. Our case demonstrates how a multidisciplinary surgical team is needed to effectively revise a VP shunt after spontaneous migration into the venous and cardiopulmonary vasculature. Further reports of shunt catheter migration would be helpful in evaluating other surgical approaches for catheter revision since no standard of care currently exists.
